# Implantation of Hypoxia-Induced Mesenchymal Stem Cell Advances Therapeutic Angiogenesis

**DOI:** 10.1155/2022/6795274

**Published:** 2022-03-20

**Authors:** Farina Mohamad Yusoff, Ayumu Nakashima, Ki-ichiro Kawano, Masato Kajikawa, Shinji Kishimoto, Tatsuya Maruhashi, Naoki Ishiuchi, S. Fadilah S. Abdul Wahid, Yukihito Higashi

**Affiliations:** ^1^Department of Cardiovascular Regeneration and Medicine, Research Institute for Radiation Biology and Medicine, Hiroshima University, Hiroshima, Japan; ^2^Department of Stem Cell Biology and Medicine, Hiroshima University Graduate School of Biomedical Sciences, Hiroshima, Japan; ^3^Division of Regeneration and Medicine, Medical Center for Translational and Clinical Research, Hiroshima University Hospital, Hiroshima, Japan; ^4^Department of Cardiovascular Medicine, Hiroshima University Graduate School of Biomedical Sciences, Hiroshima, Japan; ^5^Center for Cause of Death Investigation Research, Hiroshima University Graduate School of Biomedical Sciences, Hiroshima, Japan; ^6^Pusat Terapi Sel (Cell Therapy Centre), Universiti Kebangsaan Malaysia (UKM) Medical Centre, Kuala Lumpur, Malaysia

## Abstract

Hypoxia preconditioning enhances the paracrine abilities of mesenchymal stem cells (MSCs) for vascular regeneration and tissue healing. Implantation of hypoxia-induced mesenchymal stem cells (hi-MSCs) may further improve limb perfusion in a murine model of hindlimb ischemia. This study is aimed at determining whether implantation of hi-MSCs is an effective modality for improving outcomes of treatment of ischemic artery diseases. We evaluated the effects of human bone marrow-derived MSC implantation on limb blood flow in an ischemic hindlimb model. hi-MSCs were prepared by cell culture under 1% oxygen for 24 hours prior to implantation. A total of 1 × 10^5^ MSCs and hi-MSCs and phosphate-buffered saline (PBS) were intramuscularly implanted into ischemic muscles at 36 hours after surgery. Restoration of blood flow and muscle perfusion was evaluated by laser Doppler perfusion imaging. Blood perfusion recovery, enhanced vessel densities, and improvement of function of the ischemia limb were significantly greater in the hi-MSC group than in the MSC or PBS group. Immunochemistry revealed that hi-MSCs had higher expression levels of hypoxia-inducible factor-1 alpha and vascular endothelial growth factor A than those in MSCs. In addition, an endothelial cell-inducing medium showed high expression levels of vascular endothelial growth factor, platelet endothelial cell adhesion molecule-1, and von Willebrand factor in hi-MSCs compared to those in MSCs. These findings suggest that pretreatment of MSCs with a hypoxia condition and implantation of hi-MSCs advances neovascularization capability with enhanced therapeutic angiogenic effects in a murine hindlimb ischemia model.

## 1. Introduction

About 30% of patients with chronic threatening limb ischemia (CTLI), also known as critical limb ischemia (CLI), are still at high risk for amputation despite advances in extra-anatomical and endovascular interventions [1]. Although these approaches have enabled modest improvement in limb ischemia symptoms, improvement of the defected inflow would also require responsive outflow, especially within the microvasculature, to promote neovascularization and surrounding tissue regeneration [2–4]. It is well known that a substantial number of CTLI patients with no-option of current conventional interventions lead to a significant reduction in the quality of life with high mortality risk [4, 5].

Therapeutic angiogenesis to facilitate microvascular and surrounding tissue regeneration through various angiogenic approaches, including cell therapy and gene therapy, has been investigated in preclinical models and in clinical trials. Bone marrow mononuclear cell (BM-MNC) implantation has been shown to induce angiogenesis in both ischemic limb models and patients with limb ischemia [6–12]. BM-MNC implantation requires harvesting a large amount of BM under general anesthesia, and our recent outcome review revealed improvement in long-term major amputation-free survival [13]. However, the procedure requiring extraction of adequate BM for a therapeutic number of BM-MNCs is a burden for patients with severe complications such as myocardial ischemia, heart failure, cerebrovascular disease, and renal failure.

Mesenchymal stem cells (MSCs), which are isolated from extracted BM-MNCs, are pluripotent progenitor cells and differentiate into osteoblasts, chondrocytes, adipocytes, neurons, skeletal muscle cells, endothelial cells (ECs), and vascular smooth muscle cells. MSCs play an important role in angiogenesis and are involved in inflammatory conditions [14]. MSCs have the ability of self-renewal for ex vivo expansion, which allows repeated cell implantation to be performed, and there is no formation of carcinoma such as teratocarcinoma or hemangiosarcoma, and there is no immune rejection [14]. Repeated implantation of cells may be used as a booster strategy to acquire whole cell therapy benefits [15]. Nevertheless, the full potential of MSCs is still being examined.

Several studies have been performed to improve the capacity of MSCs to deliver their therapeutic properties by various methods including sequestration of exosomes or microvesicles, application of a conditioned medium, and hypoxia induction in cell culture [16–19]. The microenvironment of MSCs in tissue depots is characterized by a considerably low oxygen (O_2_) partial pressure, indicating that MSCs might have some resistance to an oxygen limitation [18]. It has been recognized that hypoxia activates many stress and survival pathways in MSCs. The exposure time for hypoxia-induced MSCs (hi-MSCs) in studies varied from 0 to 72 hours with an oxygen concentration from 0% to 5%. Inhibition of the proliferation of human BM-MSCs and murine adipose-derived MSCs was previously observed after 48 hours of incubation at 1% O_2_ and with serum deprivation. Nevertheless, with hypoxic preconditioning from 3 to 24 hours, the short-term stress to MSCs evoked a compensatory mechanism that elicits various signaling and metabolic pathways for cell survival, proliferation, differentiation, and migration capabilities.

Enhancement of MSC proficiency for vascular regeneration and tissue healing by hypoxia preconditioning has been reported [20–22]. The secretion of a number of angiogenic factors including vascular endothelial growth factor (VEGF) and hepatocyte growth factor (HGF) was intensified when cultured in a 1% O_2_ environment [17]. It is expected that implantation of hi-MSCs will improve limb perfusion in a murine model of hindlimb ischemia. The purpose of this study was to evaluate the effectiveness of implantation of hi-MSCs as a modality for improving therapeutic outcomes in a limb ischemic-induced model.

## 2. Materials and Methods

### 2.1. Animal Model

**Figure 1:**
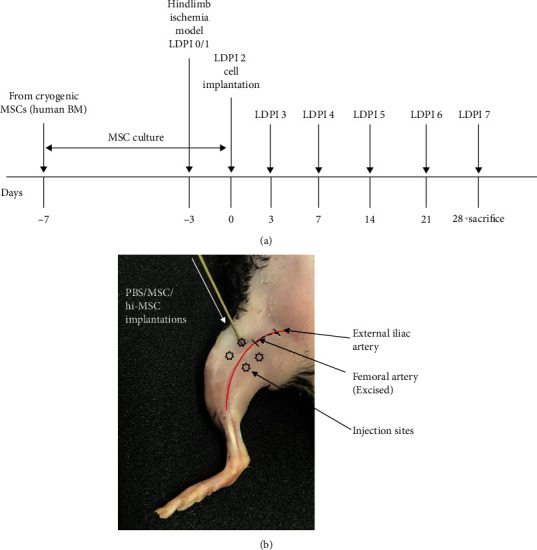
Schedule of the study and hindlimb ischemic model. (a) Protocol schedule for the in vivo disease model, mesenchymal stem cell (MSC) implantations, follow-up, and acquisition of samples. (b) Hindlimb ischemic model and phosphate-buffered saline (PBS)/MSC/hypoxia-induced MSC (hi-MSC) injection sites (stars).

Nine-week-old male C57BL/6J mice were purchased from Charles River Laboratories International, Inc. (Tokyo, Japan). The mice were housed in a light- and temperature-controlled room with normal chow diet in the Laboratory Animal Center of Hiroshima University (Hiroshima, Japan). The mice (at 10 weeks of age) were anesthetized with isoflurane delivered at ≈2% to perform ligation and excision of the right femoral artery [23]. The protocol schedule and ischemic hindlimb model diagram of the study are shown in Figures 1(a) and 1(b), respectively. The left hindlimbs of mice were left intact and used as nonischemic control limbs. After the procedure, mice were returned to their cages and allowed food and water *ad libitum*. All animal studies were conducted according to the “Guide for the Care and Use of Laboratory Animals, 8^th^ ed., 2010” (National Institutes of Health, Bethesda, MD, USA) and approved by the Institutional Animal Care and Use Committee of Hiroshima University (Permit number: A18–120).

### 2.2. Preparation of MSCs

Human MSCs isolated from bone marrow of the ilium were provided by RIKEN BRC (Ibaraki, Japan). Cryogenic BM-MSCs were seeded and grown to 60–80% confluence with fresh Dulbecco's Modified Eagle's medium (DMEM) containing 10% fetal bovine serum (FBS) every 48 hours, and the cells were later cultured under hypoxic (1% O_2_ as hi-MSCs) or normoxic (21% O_2_ as MSCs) conditions 24 hours prior to the cell implantation procedure.

### 2.3. Hypoxic Preconditioning for hi-MSCs

To perform hypoxic preconditioning, at 80% confluence, the fresh complete medium was replaced and hypoxic preconditioning was performed with a Modular Incubator Chamber (MIC 101) (Billups-Rothenberg, San Diego, CA, USA). The cells were incubated in a hypoxic condition (1% O_2_) for 24 hours.

### 2.4. Cell Viability and Proliferation Assays

Cell viability and proliferation of MSCs were analyzed by a water-soluble tetrazolium salt- (WST-) 1 assay (Takara Bio, Shiga, Japan). MSCs (2.5 × 10^3^ cells/100 *μ*L) were seeded in 96-well microplates and cultured in DMEM containing 10% FBS under normoxic (21% O_2_) or hypoxic (1% O_2_) conditions. After incubation for 0, 12, and 24 hours, 10 *μ*L WST-1 reagent was added to each well, followed by incubation for 4 hours. Absorbance was determined using a microplate reader at a wavelength of 450 nm and reference wavelength of 620 nm.

### 2.5. MSC Implantation

Injection of cultured hi-MSCs, MSCs, or phosphate-buffered saline (PBS) was performed under anesthesia 3 days after the hindlimb ischemic model had been made (Figures 1(a) and 1(b)). Twenty-four mice were randomly divided into 3 injection groups. In each group, a total of 1 × 10^5^ hi-MSCs in PBS, 1 × 10^5^ MSCs in PBS, or PBS only (as control group) with injection of 1 *μ*L per site was injected into the ischemic thigh muscle using a 24-gauge needle at four different points (Figure 1(b)). In our preliminary study, we confirmed that implantation of 1 × 10^5^ MSCs induced angiogenesis and improved blood flow perfusion in the hindlimb ischemia model. All of the mice survived without severe systemic adverse reactions after cell implantations throughout the study protocol schedule.

### 2.6. Laser Doppler Perfusion Imaging

Animals were anesthetized with isoflurane ≈ 2% for laser Doppler perfusion imaging (LDPI). The images for measurements were taken before the hindlimb ischemia model procedure, immediately after the procedure and on days 3, 7, 14, 21, and 28 after implantation. At each time point, tissue perfusion was measured by imaging the blood flow in both the ischemic and nonischemic limbs. The results were reported as the ratio of those two measurements. Mice were sacrificed by cervical dislocation under deep anesthesia for harvest of ischemic and nonischemic limb muscle tissues at day 28 after implantation for analyses.

### 2.7. Immunofluorescence Staining

Immunofluorescence staining was performed to detect the expression of EC markers (cluster of differentiation 31, CD31) in ischemic hindlimb muscles after cell implantation. At day 28 after cell implantation, muscle tissue was harvested and snap-frozen using 2-methylbutane and liquid nitrogen. In brief, 5 *μ*m muscle tissue sections were prepared by transversal cryosectioning and then mounted onto glass slide and air-dried. The sections were then fixed with 4% paraformaldehyde and blocked with Block ACE (DS pharma Biomedical Co., Ltd., Osaka, Japan). Tissue slices were incubated with primary antibody CD31 (1 : 200) overnight at 4°C. AlexaFluor-conjugated secondary antibody (1 : 500) (Invitrogen Corporation, Carlsbad, CA) was then added. Cell nuclei were also counterstained with 4′,6-diamidino-2-phenylindole (DAPI). CD31-positive cells were imaged using microscopy and image software (KEYENCE Co., Ltd., Osaka, Japan).

### 2.8. Western Blot Analysis

The expression levels of angiogenic cytokines in cultured MSCs and hi-MSCs prior to implantation were analyzed. In brief, the cells were homogenized in a lysis buffer and then centrifuged to extract the lysate. The lysate was then used for western blot analysis of hypoxia-inducible factor-1 alpha (HIF-1*α*), vascular endothelial growth factor A (VEGF-A), and peroxisome proliferator-activated receptor *γ* coactivator-1*α* (PGC-1*α*) proteins. Primary antibodies used in this study were monoclonal anti-HIF-1*α* antibody (Cell Signaling Technology, Danvers, MA, USA), polyclonal anti-VEGF-A antibody (Abcam, Cambridge, UK), and polyclonal anti-PGC1-*α* antibody (Abcam, Cambridge, UK). The expression levels of these proteins were quantified in comparison with the amount of beta actin (Abcam, Cambridge, UK).

### 2.9. Endothelial Differentiation of MSCs

**Figure 2:**
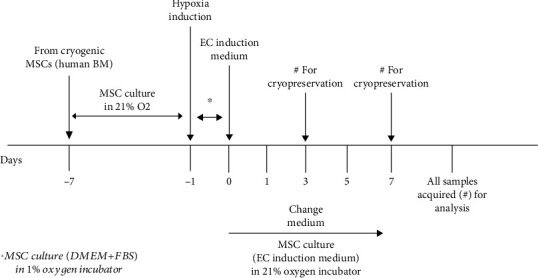
Protocol schedule for in vitro endothelial cell (EC) differentiation challenge in mesenchymal stem cells (MSCs) vs. hypoxia-induced MSC (hi-MSC) models, follow-up, and acquisition of samples at day 3 and day 7 after application of EC-induction medium.

EC-induction medium was used to isolated MSCs and hi-MSCs according to a previously reported protocol with some modifications [24]. In brief, an induction medium containing Iscove's Modified Dulbecco's Medium (IMDM), 2% FBS, 1% P/S, L-glutamine, 50 ng/mL VEGF, 10 ng/mL bFGF, 20 ng/mL IGF, 5 ng/mL EGF, and ascorbic acid was used (Figure 2). To confirm the endothelial phenotype, the induced cells were harvested and analyzed for EC-specific markers using quantitative real-time PCR (qRT-PCR). Assessments with gene expression assays were performed for von Willebrand factor (vWF), vascular endothelial cadherin (VE-cadherin), and vascular endothelial growth factor receptor-2 (VEGFR-2) genes to ensure the presence of differentiated ECs.

### 2.10. Quantitative Reverse Transcription Real-Time Polymerase Chain Reaction (qRT-PCR)

Total RNA extraction, synthesis of cDNA, and real-time reverse transcription polymerase chain reaction (PCR) were performed on induced cells. Results of PCR experiments were analyzed by TaqMan Gene Expression Assays and SYBR Gene Expression Assays with 7500 Fast (Applied Biosystems, Foster City, CA). Specific oligonucleotide primers and probes for human VEGF (assay ID: Hs00900055_m1) and platelet endothelial cell adhesion molecule-1 (PECAM-1) (assay ID: Hs01065279_m1) were obtained for TaqMan Gene Expression Assays (Applied Biosystems, Foster City, CA, USA), and an SYBR Green RT-PCR Kit (Life, Foster City, CA, USA) was used to detect the expression of target genes of vWF with 5′-CCCATTTGCTGAGCCTTGT-3′ (forward) and 5′-GGATGACCACCGCCTTTG-3′ (reverse). The mRNA levels of the samples were normalized to the level of GAPDH, and the relative expression level of each target gene was calculated by *ΔΔ*Ct.

### 2.11. Endothelial Cell Migration (Coculture) Assays

Monocultures of MSCs and hi-MSCs, coculture of MSCs with human umbilical vein endothelial cells (HUVECs) (MSC coculture), and coculture of hi-MSCs with HUVECs (hi-MSC coculture) were analyzed at the 3^rd^ hour (3 hr), 6^th^ hour (6 hr), and 10^th^ hour (10 hr) using the BD Falcon™ Insert System for the cell migration study. The 3 *μ*m and 8 *μ*m pore-sized BD Insert Systems were used, and cells were incubated in Basal Media+EGM™-2 Endothelial SingleQuots™ Kit (Cat No.: CC-4176) at 5% CO2 and 37°C. Seeding density of MSCs, hi-MSCs, and HUVECs was about 50,000 cells/well in a 24-well uncoated insert system and with a ratio of 1 : 1 for the coculturing. Cells were stained with a CD31 antibody (CD31+) using the DAB/Hematoxylin (brown; diaminobenzidine, DAB) method, and the stained cells were observed by using a bright-field inverted microscope. Each experiment was performed 4 times.

### 2.12. Statistical Analysis

Values are presented as categorical variables and means ± SE. Variables were compared by using the Tukey-Kramer HSD between 3 groups and Student's *t*-test between 2 groups. Values of *P* < 0.05 were considered significant. Data were processed using the JMP version 15.0 software (SAS Institute, Cary, NC, USA).

## 3. Results and Discussion

### 3.1. Hypoxic Preconditioning Enhanced the Cell Viability and Proliferation of MSCs

**Figure 3:**
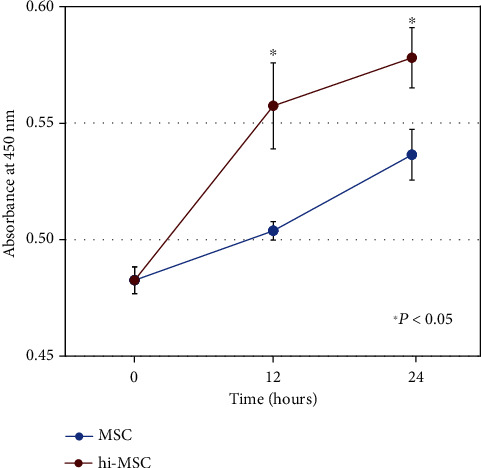
Cell viability and proliferation assays. The absorbance value of hypoxia-induced MSCs (hi-MSCs) was more significantly increased than that of mesenchymal stem cells (MSCs) in a time-dependent manner. The absorbance value of MSCs (*n* = 5) and hi-MSCs (*n* = 5) was assessed at each time point (0, 12, and 24 hours).

HIF plays an important role in MSC physiology under hypoxic conditions by controlling the metabolic fate and multipotency of MSCs [17, 18]. HIF-1*α* influences the colony-forming mesenchymal progenitors to promote self-renewal of the MSC population. A specific hypoxia precondition of MSCs has been found to enhance the cell viability and proliferation of MSCs. Nevertheless, there are reports of both upregulation and downregulation of proliferation or differentiation of MSCs depending on the type of protocol, culture medium composition, oxygen tension applied, duration of hypoxia treatment, and also the heterogeneity of donors [25]. Therefore, we assessed the cell viability and proliferation of hi-MSCs using a WST-1 assay and found that the absorbance value of hi-MSCs was more significantly increased than that of MSCs in a time-dependent manner (Figure 3), indicating that hypoxic preconditioning enhanced the cell viability and proliferation of MSCs.

### 3.2. hi-MSC Implantation Improves Angiogenesis

**Figure 4:**
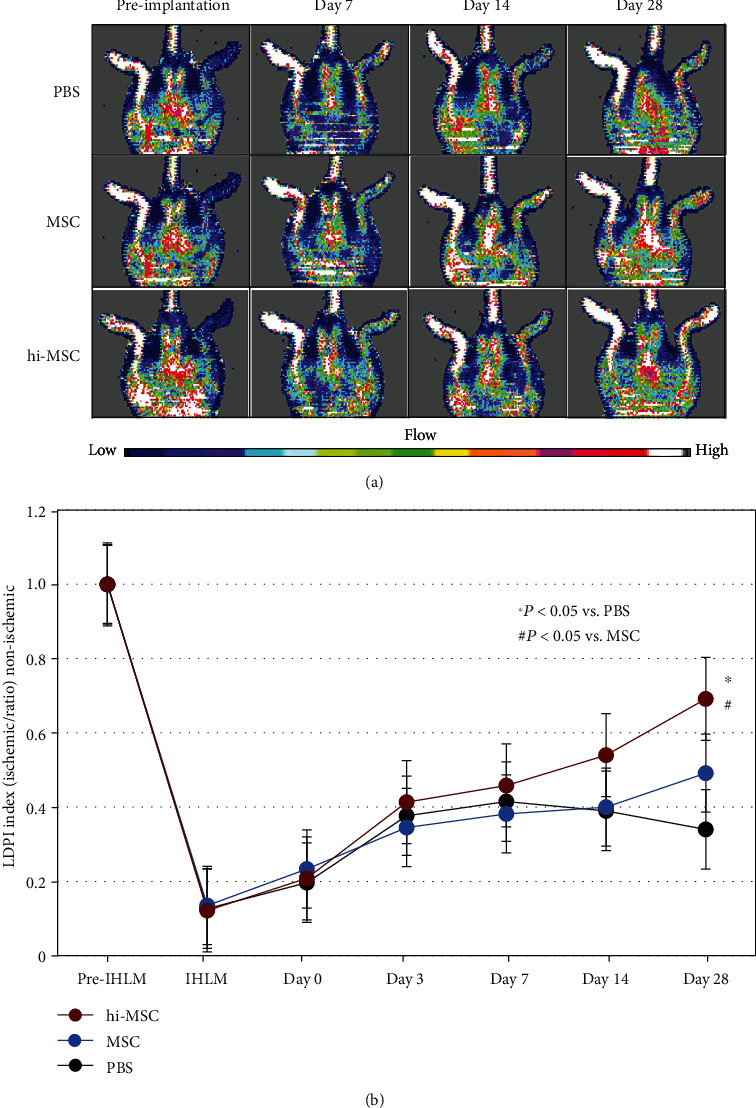
Serial laser Doppler perfusion images (LDPIs) before and after making the ischemic hindlimb model. (a) Representative images of LDPI studies. (b) LDPI index studies in phosphate-buffered saline (PBS; *n* = 6), mesenchymal stem cell (MSC; *n* = 6), and hypoxia-induced MSC (hi-MSC; *n* = 6) implantation groups.

Limb perfusion by serial LDPI study before and after making the ischemic hindlimb model was performed in mice as shown in Figures 4(a) and 4(b).

At day 7 after cell implantation, there were no significant differences in LDPI index among the three groups. At day 28, LDPI index was significantly higher in the hi-MSC group than in the PBS and MSC groups, while there was no significant difference in LDPI index between the PBS and MSC groups. These findings suggest that hi-MSC implantation significantly improved blood flow recovery after hindlimb ischemia compared to PBS and MSC implantation (*P* < 0.05, respectively).

There was significant limb perfusion recovery with hi-MSC implantation performed 3 days after the ischemic hindlimb model had been made. Immediately after ischemia, the hypoxic environment in muscle tissue triggers postischemia angiogenesis. Under a hypoxic condition, HIF-1*α* becomes rapidly activated, resulting in increased gene expression of proangiogenic factors such as VEGF, fibroblast growth factor (FGF), and stroma-derived factor-1 alpha (SDF-1*α*). Subsequently, VEGF induces angiogenesis, characterized by capillary sprouting from preexisting blood vessels, EC migration, proliferation, and luminogenesis to generate new capillaries. Vasculogenesis and arteriogenesis are also initiated through the recruitment of endothelial progenitor cells (EPCs), which express a range of proangiogenic cytokines and growth factors including VEGF, HGF, FGF, angiopoietin-1, SDF-1*α*, insulin-like growth factor-1, and endothelial nitric oxide synthase (eNOS)/inducible NOS (iNOS) [14, 26–28]. The presence of expanded neovascularization due to the presence of effective angiogenic factors in the skeletal muscle microvasculature has been shown to improve blood flow and blood perfusion.

**Figure 5:**
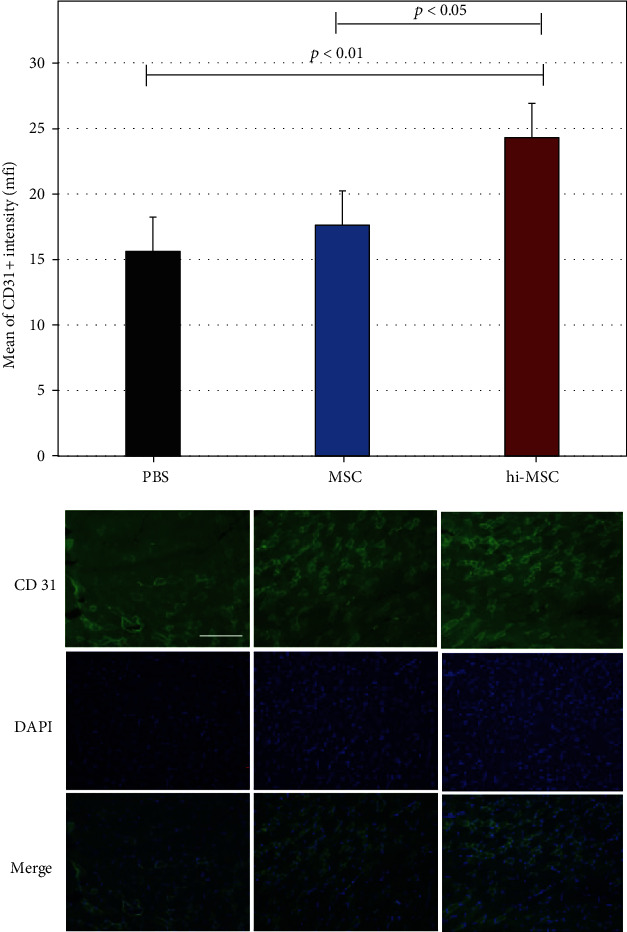
Mean cluster of differentiation 31 (CD31) intensity at day 28 after surgical induction of ischemia. (a) Mean CD31 intensity in phosphate-buffered saline (PBS; *n* = 6), mesenchymal stem cell (MSC; *n* = 6), and hypoxia-induced MSC (hi-MSC; *n* = 6) injection groups. (b) Representative images of CD31 immunostaining. Scale bar: 50 *μ*m.

As shown in Figure 5, the mean CD31 intensity in ischemic muscles at day 28 was greater in the hi-MSC group than in the PBS and MSC groups (*P* < 0.01 and *P* < 0.05, respectively). Blood flow recovery after hindlimb ischemia was also slightly improved in the MSC group compared to that in the PBS group. With a significantly higher expression level of CD31 in the hi-MSC group than in the PBS and MSC groups, hi-MSC implantation significantly improved blood flow recovery, suggesting that implantation of hi-MSCs promotes angiogenesis after induction of hindlimb ischemia.

The skeletal muscle is a highly vascularized organ containing the highest microvascular mass and is highly adaptable, responding to environmental and physiological demands that control and maintain bodily functions for survival [29, 30]. Conservation of the microcirculation structure emphasizes a major role of capillary-muscle interactions in physiological conditions. Diabetes mellitus and chronic renal diseases are associated with skeletal muscle dysfunction and microvasculature changes that can lead to degradation of the vascular network. Neovascularization in the microenvironment for skeletal muscle remodeling and recovery from pathologies seem to be essential to establish promising therapeutic strategies. This study has shown that effective microvascular regeneration can be achieved by hi-MSC implantation than by only MSC implantation. The significantly increased presence of ECs in the hi-MSC implantation group showed an efficient neovascularization for improvement in blood flow and blood perfusion. It is important that recovery of the vascular network after ischemia occurs within the microvascular unit of the skeletal muscle.

### 3.3. Expression of Angiogenic Factors in hi-MSCs and MSCs Before Implantation

**Figure 6:**
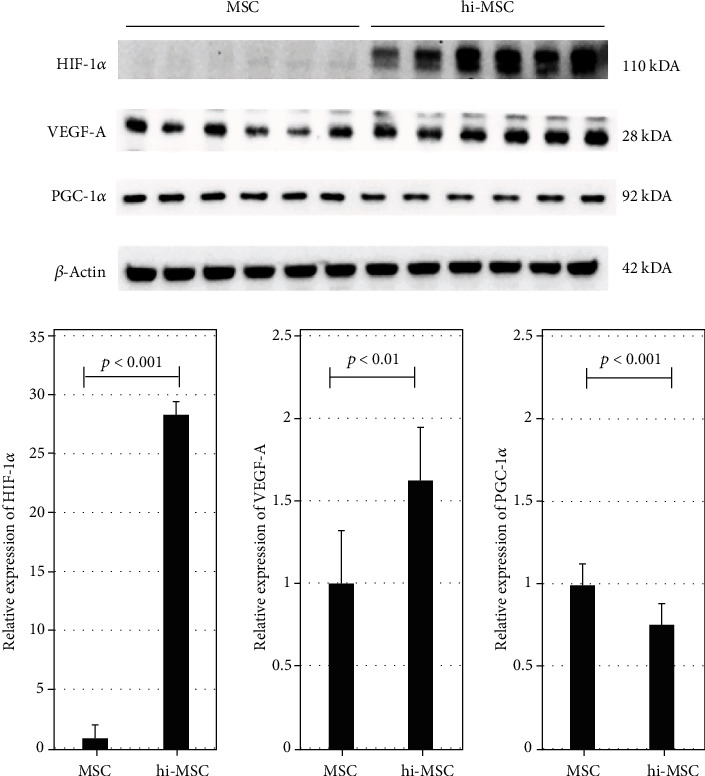
Expression of angiogenic cytokines in cultured mesenchymal stem cells (MSCs) and hypoxia-induced MSCs (hi-MSCs) prior to implantation. hi-MSCs showed significantly higher expression levels of hypoxia-inducible factor-1 alpha (HIF-1*α*) and vascular endothelial growth factor A (VEGF-A) than those in MSCs. The expression level of peroxisome proliferator-activated receptor *γ* coactivator-1*α* (PGC-1*α*) was slightly lower in the hi-MSC group than in the MSC group.

The expressions levels of HIF-1*α* and VEGF-A were significantly higher in hi-MSCs than in MSCs (Figure 6). There was a slightly but significantly lower expression level of PGC-1*α* in hi-MSCs than in MSCs. Along with these differences in expression levels of HIF-1*α*, VEGF-A, and PGC-1*α* prior to cell implantation into ischemic muscles, different angiogenesis outcomes were found among the in vivo groups.

The key adaptive response to hypoxic conditions is stabilization of HIF-1 [26]. As previously reported, angiogenesis is triggered by reduced oxygen delivery to the tissue and is mainly under the control of the transcriptional factor HIF-1*α* generated by numerous cells in the ischemic tissue. HIF-1 accumulation and degradation through multiple signaling pathways lead to effective tissue recovery and maintenance. HIF-1*α* and VEGF are potent triggers for postnatal, postischemia neovascularization. As the increase in O_2_ levels following early neovascularization, HIF-1*α* is rapidly degraded by the proteasome. Oxygen regulates the degradation process by the addition of hydroxyl groups to HIF-1*α*. Prolonged hypoxia and inflammation cause maladaptation of HIF-1 that can lead to ineffective VEGF regulation and impair microvascular regeneration and tissue recovery.

PGC-1*α* is one of important regulators of oxidative metabolism and mitochondrial function and is also involved in induction of VEGF [23, 31, 32]. Overexpression of PGC-1*α* enhances the survival and angiogenic potential of MSCs, and transplantation of MSCs modified with PGC-1*α* can result in significantly greater improvement in diabetic hindlimb ischemia than that after transplantation of MSCs. In this study, PGC-1*α* was present in both MSCs and hi-MSCs and limb blood flow and perfusion were enhanced. Nevertheless, despite slightly reduced expression of PGC-1*α* in the hi-MSC population of cells, the potent effects of HIF-1*α* and VEGF enabled advancement of neovascularization in the studied samples.

### 3.4. EC-Differentiation Capacity of hi-MSCs and MSCs

**Figure 7:**
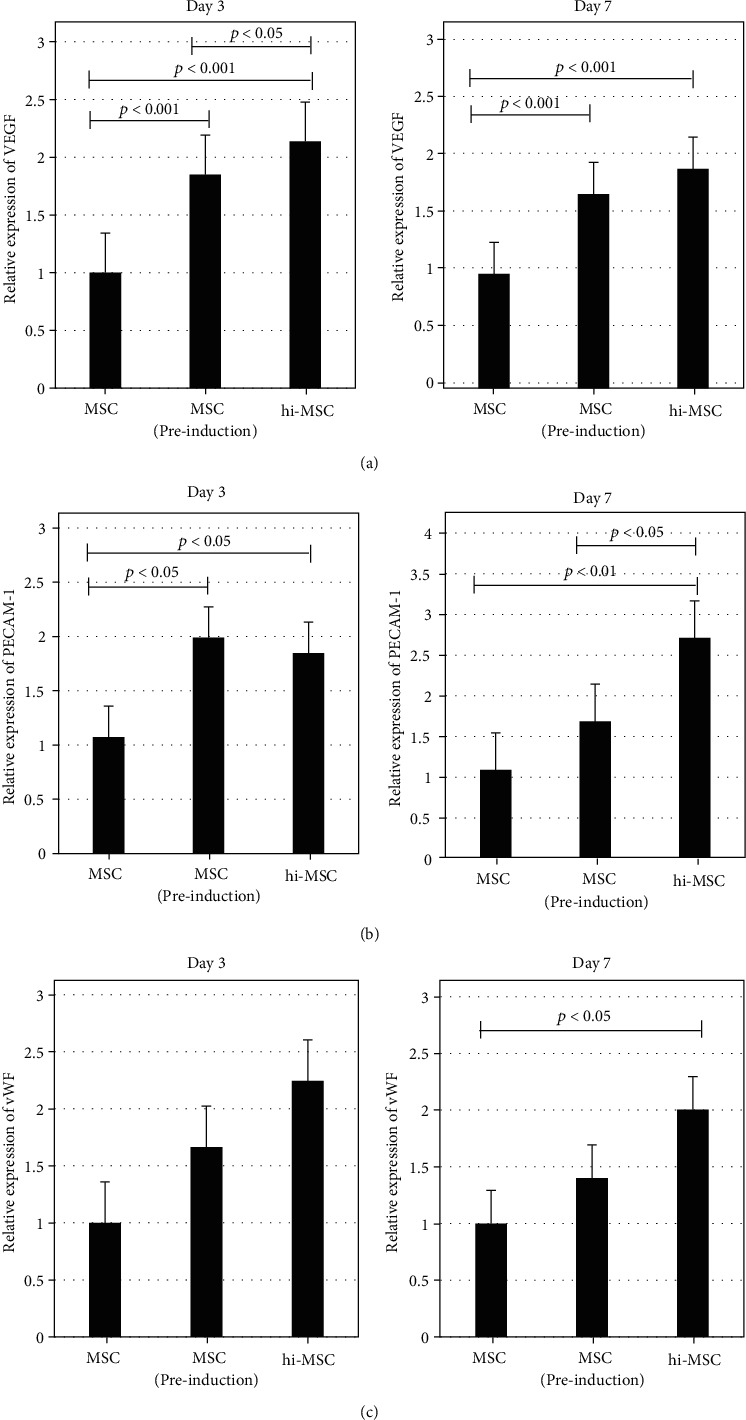
Endothelial cell (EC) differentiation challenge of mesenchymal stem cells (MSCs) and hypoxia-induced MSCs (hi-MSCs) with analysis through TaqMan and SYBR Gene Expression Assays at day 3 and day 7 after cells were cultured with EC-induction medium. (a) Analysis of vascular endothelial growth factor (VEGF) gene expression. (b) Analysis of platelet endothelial cell adhesion molecule-1 (PECAM-1) gene expression. (c) Analysis of von Willebrand factor (vWF) gene expression. hi-MSC population of cells may have added potential for EC differentiation compared to the MSC population of cells.

To determine the EC-differentiation capacity of MSCs, we investigated the expression levels of EC markers in MSC and hi-MSC populations of cells after application of an induction medium (Figure 2). Samples were analyzed on the day before application of the induction medium and on day 3 and day 7 after application of the induction medium. qRT-PCR revealed that the expression levels of vWF, VE-cadherin, and VEGFR-2 were significantly higher in the induced MSC group than in the uninduced MSC group (Figures 7(a)–7(c)).

MSCs have been reported to undergo endothelial differentiation [24, 33–35]. Under a condition of reduced oxygen, MSCs display heightened angiogenic activity. Previous studies have shown upregulation of the expression of “stemness” genes including Oct-4, Rex-1, STRO-1, Sca-1 + and Sca-1 +/CD44 +, Sox2, Nanog, and SSEA-4, suggesting that MSCs under a hypoxic condition have the properties of true stem cells [18]. At 1% O_2_, murine BM-MSCs rapidly migrate and form three-dimensional capillary-like structures in matrigel, synthesize more VEGF, and downregulate matrix metalloproteinase- (MMP-) 2 mRNA expression and protein secretion, while the expression of membrane-type 1-MMP is strongly induced by hypoxia. Hypoxia can increase self-renewal, multipotency, and the transdifferentiation potential of MSCs.

Asahara et al. demonstrated that circulating EPCs may contribute to reendothelialization of the injured endothelium and neovascularization at sites of ischemia [36, 37]. EPCs are derived from bone marrow, and these cells differentiate into vascular ECs. In animal models of ischemia, heterologous, homologous, and autologous EPCs were incorporated into the sites of active angiogenesis. These findings suggest that EPCs may be useful for augmenting growth of collateral vessels to ischemic tissues as therapeutic angiogenesis and for delivering antiangiogenic or proangiogenic agents to sites of pathologic angiogenesis. Shintani *et al*. reported that the use of BM-MNCs, including EPCs, was sufficient and effective for therapeutic angiogenesis in rabbit models of limb ischemia [6]. Our data suggest that a small population of EPCs may be present in the MSC population of cells and have better capacity in EC-differentiation in a hypoxic environment. Hence, these circumstances can be a contributing factor to improvement of neovascularization in hi-MSCs compared to the MSC population of cells.

After ischemia, numerous cells elicit immediate inflammatory and angiogenesis responses to stabilize the disrupted system. Overt inflammation and prolonged hypoxia have been shown to impair and delay optimum tissue recovery. The interplay between MSCs, inflammation, and angiogenesis is important at the early stage and during subsequent progression of tissue healing [17, 38, 39]. Intramuscular delivery with precision engraftment of cells into maladaptive ischemic tissue can facilitate recovery after the initial phase of tissue injury [8, 40, 41]. Multiple modality and paracrine functions of MSCs are further improved by hypoxia preconditioning.

### 3.5. Endothelial Cell Migration Assays (Coculturing)

**Figure 8:**
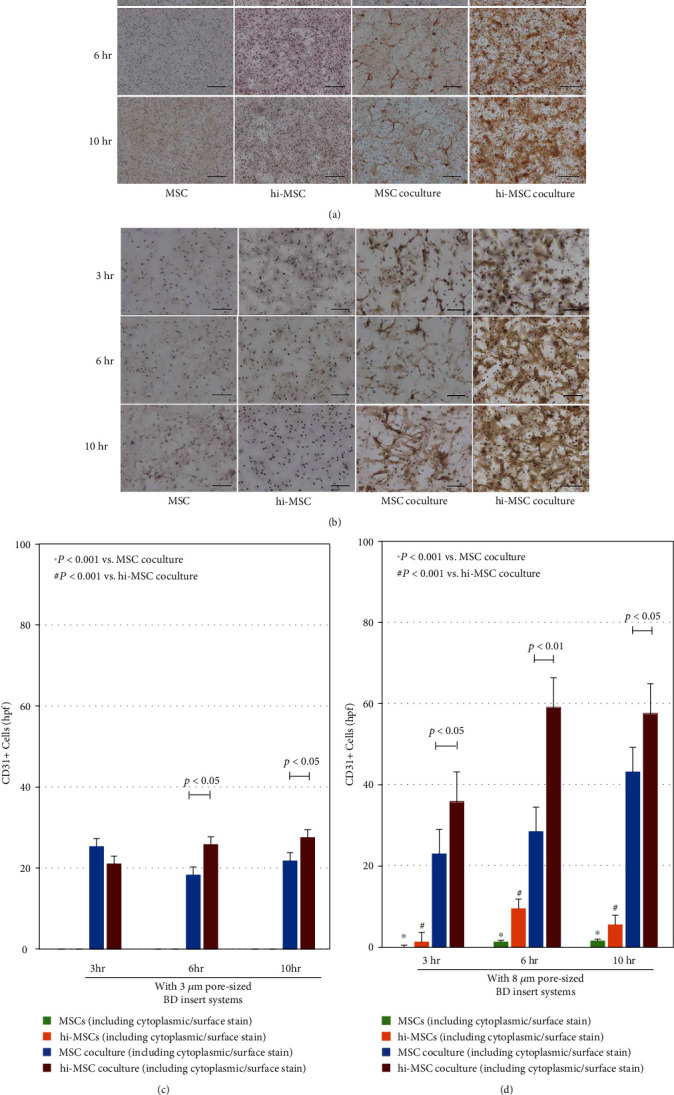
In vitro cell migration assays of mesenchymal stem cells (MSCs), hypoxia-induced MSCs (hi-MSCs), coculture of MSCs with human umbilical vein endothelial cells (HUVECs) (MSC coculture), and coculture of hi-MSCs with HUVECs (hi-MSC coculture) at each time point of the 3^rd^ hour (3 hr), 6^th^ hour (6 hr), and 10^th^ hour (10 hr) with (a, c) 3 *μ*m pore-sized inserts and (b, d) 8 *μ*m pore-sized. The hi-MSC population of cells may have added potential for coculture migration capacity compared to the MSC population of cells. Scale bar: 50 *μ*m.

We proceeded to investigate the potential of hi-MSCs in influencing the migration of endothelial cells. We performed coculture of MSCs with HUVECs (MSC coculture), coculture of hi-MSCs with HUVECs (hi-MSC coculture), and monocultures of MSCs and hi-MSCs for comparative assessments at each time point of the 3^rd^ hour (3 hr), 6^th^ hour (6 hr), and 10^th^ hour (10 hr) (Figures 8(a) and 8(b)). The migratory capacity of hi-MSC coculture with HUVECs was found to be significantly greater than that in MSC coculture (*P* < 0.01 and *P* < 0.05, respectively) (Figures 8(c) and 8(d)). Without the presence of HUVECs, we found that there was no significant cell migration when using the 3 *μ*m pore-sized inserts (Figures 8(a) and 8(c)), whereas a small number of MSCs migrated when 8 *μ*m pore-sized inserts were used (Figures 8(b) and 8(d)). The migratory capacity of CD31+ cells in MSC coculture was significantly lower than that in hi-MSC coculture and was consistent across time points. hi-MSCs may have the capacity to influence the migration of endothelial cells. Multiple publications have reported that HIF-1*α* is involved in the proliferation, migration, and differentiation of endothelial cells [19, 26, 42]. Hirato and Semenza discussed the importance of HIF-1-regulated factors in the steps in angiogenesis that include arterial destabilization, increased vascular permeability, extracellular matrix remodeling, migration and proliferation of endothelial cells, endothelial cell sprouting, tube formation and cell-to-cell contact, recruitment of and interaction with pericytes, and maintenance of vessel integrity. Those factors include EGF, PLGF, Flt-1, MMPs, VEGF, MCP-1, PDGF, SDF-1, CXCR4, integrins, PDGF, PAI-1, angiopoietin-1, angiopoietin-2, and Tie-2. By promoting angiogenesis through proliferation, migration, and differentiation of endothelial cells, those factors that are regulated by HIF-1 have led to the improvement of blood flow recovery after hindlimb ischemia. By reintroducing HIF-1*α* and VEGF through hi-MSCs, the other angiogenic factors are further regulated to enable more processes of angiogenesis to occur during the strategic therapeutic cells' implantations.

## 4. Conclusion

Pretreatment of MSCs with a hypoxia condition and intramuscular implantation of hi-MSCs advances neovascularization capability with enhanced therapeutic angiogenic effects in a murine hindlimb ischemia model. Implantation of hi-MSCs promotes angiogenesis and augments neovascularization with the presence of higher expression levels of angiogenic cytokines including HIF-1*α* and VEGF. Future studies are needed to determine the role of hi-MSCs in therapeutic angiogenesis in order to confirm the efficacy, safety, and feasibility of MSC implantation for long periods.

## Data Availability

The dataset used to support the findings of this study are available from the corresponding author upon request.
